# The Behavioural Inhibition System, anxiety and hippocampal volume in a non-clinical population

**DOI:** 10.1186/2045-5380-4-4

**Published:** 2014-03-07

**Authors:** Liat Levita, Catherine Bois, Andrew Healey, Emily Smyllie, Evelina Papakonstantinou, Tom Hartley, Colin Lever

**Affiliations:** 1 Present address: Department of Psychology, University of Sheffield, Western Bank, Sheffield S10 2TN, UK; 2 Department of Psychology, University of York, York YO10 5DD, UK; 3 Department of Psychiatry, University of Edinburgh, Edinburgh EH10 5HF, UK; 4 Department of Psychology, University of Durham, Durham DH1 3LE, UK

**Keywords:** Anxiety, Behavioural Inhibition System, Sensitivity to Punishment, Structural MRI, Hippocampus, Amygdala

## Abstract

**Background:**

Animal studies have suggested that the hippocampus may play an important role in anxiety as part of the Behavioural Inhibition System (BIS), which mediates reactivity to threat and punishment and can predict an individual’s response to anxiety-relevant cues in a given environment. The aim of the present structural magnetic resonance imaging (MRI) study was to examine the relationship between individual differences in BIS and hippocampal structure, since this has not received sufficient attention in non-clinical populations. Thirty healthy right-handed participants with no history of alcohol or drug abuse, neurological or psychiatric disorders, or traumatic brain injury were recruited (16 male, 14 female, age 18 to 32 years). T1-weighted structural MRI scans were used to derive estimates of total intracranial volume, and hippocampal and amygdala gray matter volume using FreeSurfer. To relate brain structure to Gray’s BIS, participants completed the Sensitivity to Punishment questionnaire. They also completed questionnaires assessing other measures potentially associated with hippocampal volume (Beck Depression Inventory, Negative Life Experience Survey), and two other measures of anxiety (Spielberger Trait Anxiety Inventory and the Beck Anxiety Inventory).

**Results:**

We found that high scores on the Sensitivity to Punishment scale were positively associated with hippocampal volume, and that this phenomenon was lateralized to the right side. In other words, greater levels of behavioural inhibition (BIS) were positively associated with right hippocampal volume.

**Conclusions:**

Our data suggest that hippocampal volume is related to the cognitive and affective dimensions of anxiety indexed by the Sensitivity to Punishment, and support the idea that morphological differences in the hippocampal formation may be associated with behavioural inhibition contributions to anxiety.

## Background

Lang’s tripartite model of anxiety suggests that it consists of three response domains: cognitive, behavioural, and physiological [1], which together result in a state of apprehensive worry, hyperarousal to threat cues, avoidance behaviours and negatively-biased cognitions [2]. Each of these domains is suggested to measure a separate element of response characteristics and potentially independent underlying mechanisms to the construct of anxiety [3]. An influential model of anxiety sees it as reflecting the engagement of the Behavioural Inhibition System (BIS) of which the hippocampus is a key component [4]. Briefly, in Gray’s original account the role of BIS is to govern avoidance behaviours in response to threat and punishment. Excessive activity in BIS when driven by enhanced reactivity to threat/punishment cues manifests as higher proneness to anxiety.

In support of this idea Gray reviewed the evidence in the animal literature that anxiolytic drugs impair hippocampal function, specifically septo-hippocampal theta, to suggest that the hippocampus was the key substrate of BIS [4]. Subsequent revision of the theory has incorporated other regions, most notably the amygdala, as a part of the BIS network, with the amygdala and hippocampus mediating different aspects of anxiety [5,6], and with the BIS interpreted as a conflict mediator system biased toward fight/flight/freeze behaviours and using exploration to resolve conflict. Critically, ensuing empirical work has continued to implicate hippocampal theta in anxiety and anxiolytic drug effects (for examples, see [7-14]). For example, Gray and McNaughton [5] observe that anxiolytic drugs, despite their neurochemical dissimilarity, commonly reduce the frequency of reticular-elicited hippocampal theta in the anaesthetised animal. We recently showed that Gray and McNaughton’s central observation extends to the awake, freely moving rat, where anxiolytic drugs reduce the frequency of natural theta obtained during locomotion [14].

Two commonly used and well-validated instruments designed to measure individual differences in Gray’s BIS are the BIS section of the BIS/Behavioural Activation System scales [15] and the Sensitivity to Punishment (StP) subscale of the Sensitivity to Punishment and Sensitivity to Reward questionnaire [16]. These instruments have been shown to predict clinical anxiety disorders (for examples, see [17,18]), and likely capture cognitive and affective, rather than somatic, aspects of anxiety [16]. Using these instruments and other indicators of BIS activity, neuroimaging studies have begun to implicate the hippocampus and amygdala in behavioural inhibition. Hahn and colleagues [19] found that StP scores predicted hippocampus-amygdala functional connectivity in a monetary loss anticipation task. Further, it is conceivable that hippocampal structure, as well as activity, may be partly heritable. This is supported by a study by Oler and colleagues [20] who investigated ‘anxious temperament’ in monkeys using a three-part composite measure of anxiety consisting of two behavioural BIS measures and cortisol release. They found that anxiety was clearly heritable, and that both hippocampal and amygdalar activity predicted anxiety, but only the hippocampal anxiety-related activity was heritable.

Together these findings suggest that BIS-related anxiety may be associated with structural variations in the brain. To our knowledge, only three studies have specifically related brain volume measures to BIS self-report [21-23]. Interestingly, two of these found that (para)hippocampal volume positively correlates with behavioural inhibition, one using voxel-based morphometry (VBM) and the StP questionnaire [22], the other using volume measures based on manual tracings and the BIS scale [21]. In the VBM study, the region correlating with StP scores was largely parahippocampal, but reportedly also included the right hippocampus proper [22]. A similar but weaker correlation based on a largely middle-aged sample was found in the manual tracing study [21].

A different approach to the BIS has been to look at neural asymmetry in human scalp electroencephalography (EEG), with right brain dominance, particularly prefrontal, associating with higher behavioural inhibition [24,25] and anxiety [26-29]. Intriguingly, simply being left-handed, and thus more likely to be right-hemisphere dominant, predisposes to higher BIS activity and anxiety [30]. Hippocampal activity cannot itself be detected by scalp EEG, but animal models suggest that hippocampal influences on prefrontal EEG are important in anxiety [7].

In the present study we used an automated segmentation method to obtain gray matter volumes of both the hippocampus and amygdala in healthy adult students, with no current or past history of any mental health disorder. Restricting our sample to young, well-educated adults may be important in minimising confounding effects of depression, stress and education. Torrubia and colleagues [16] suggest that StP implements Gray’s theoretical construct of anxiety more faithfully than Carver and White’s BIS scale. Notably, for instance, in Gray’s conceptual revision of Eysenck’s theory of personality, Gray theorised that anxious people would be both ‘introverted’ and ‘neurotic’. Consistent with this prediction, StP scores are positively correlated with Neuroticism and negatively correlated with Extraversion [16], whereas scores on Carver and White’s BIS scale tend only to be positively correlated with Neuroticism [15]. Torrubia and colleagues [16] also suggest that their focus on the response to particular cues was more in line with Gray’s theory. Accordingly, in order to relate brain structure to Gray’s BIS, we asked participants to complete the StP subscale of the Sensitivity to Punishment and Sensitivity to Reward questionnaire [16]. Participants also completed questionnaires assessing other measures potentially associated with hippocampal volume: depression with the Beck Depression Inventory (BDI)-II [31], negative life events with the Life Experiences Survey (LES) [32]; and two other measures of anxiety: Trait anxiety of the State and Trait Anxiety Inventory (STAI-T) [33] and the Beck Anxiety Inventory (BAI) [34], the latter thought to be particularly sensitive to panic symptomatology [35]. These different measurement instruments approach anxiety differently, which is why we choose to use them in this study. For instance, StP likely captures cognitive and emotional, but not somatic, components of anxiety, while BAI certainly does tap the somatic component [16,35]; trait anxiety as measured by STAI-T is dissociable from anxiety as mediated by BIS [21], and may predict depression and negative affect as much as, or even more than, anxiety *per se*[36,37]. If StP were found to be significantly related to hippocampal volume, we aimed to be able to examine the potential selectivity of this relationship.

## Methods

### Participants

Thirty healthy right-handed native English speakers (16 male, 14 female, aged 18 to 32 years, (mean ± SD, 24.1 ± 2.66 years)) were recruited from the student population at the University of York. All the participants recruited had previously undergone a structural magnetic resonance imaging (MRI) scan at the York Neuroimaging Centre. Participants were scanned 0 to 2 years prior to taking part in this study (median, 188 days). None of the participants had a history of alcohol or drug abuse, neurological or psychiatric disorders, or traumatic brain injury. This was determined by a list of questions, verbally administered by the experimenter, about past and present history of drug use and mental health status. The study was approved by the York Neuroimaging Centre Research Ethics and Governance committee. All participants gave written informed consent for participation in the study.

### Procedure

Participants were invited to attend a 1-hour test session at the Psychology Department of the University of York. All self-report inventories were administered on-line using LimeSurvey. The on-line questionnaires were administered in a counterbalanced order to control for order of presentation effects. An intelligence quotient (IQ) test was administered between the on-line questionnaires.

### Measures

All participants completed the StP scale, which is a revision of the Susceptibility to Punishment scale that was first published by Torrubia and Tobena [38] designed to measure individual differences in the Behavioural Inhibition system (BIS). The StP scale is a 24-item scale, with high internal consistency (α = 0.83) and test-retest reliability coefficients ranging up to 0.85, indicating that scores on this scale are indicative of a long-lasting aspect of anxiety [16]. The items included in this version were devised to measure individual differences in functions dependent on the BIS in situations involving the possibility of aversive consequences or novelty as well as items that assess cognitive processes produced by threat of punishment of failure.

In order to obtain comparative measures of potentially different aspects of anxiety, participants also completed the BAI and the STAI-T. The BAI is a 21-item self-report inventory used to assess primarily the intensity of somatic (hands trembling, face flushed) anxiety symptoms experienced over the last week, with each item having a scale value of 0 to 3. A score of 0 to 7 is considered minimal, 8 to 15 indicates mild anxiety, 16 to 25 reflects moderate anxiety, and 26 to 63 is considered severe anxiety. The BAI scale has high internal consistency (*α* = 0.92) and high discriminant validity against depression [34]. The State and Trait Anxiety Inventory consists of both a measure of State anxiety (STAI-S) and a measure of Trait anxiety (STAI-T) [33,39]. Each scale has 20 items. The STAI-T scale has been found to have high internal consistency (*α* = 0.9) [40].

Further, all participants were matched on IQ, as measured by the Wechsler Abbreviated Scale of Intelligence-III two-test subscales, vocabulary and matrix-reasoning, respectively [41]. In addition, BDI-II was administered [31] as depression has also been shown to affect hippocampal volume (for examples, see [42,43]). Since trauma and negative life events have been shown to be positively associated with anxiety [32], participants also filled in the LES [32], where participants are required to indicate which positive and negative events listed in the survey they had experienced in the last year. Our sample experienced very low levels of negative life events (range 1 to 27), and scores of negative life events were not correlated with StP scores (r = -0.162, *P* = 0.144), or any other of our measures of emotionality. None of these psychometric measures correlated with age, except BAI (see Additional file 1: Table S1).

### Automated segmentation analysis

**Figure 1:**
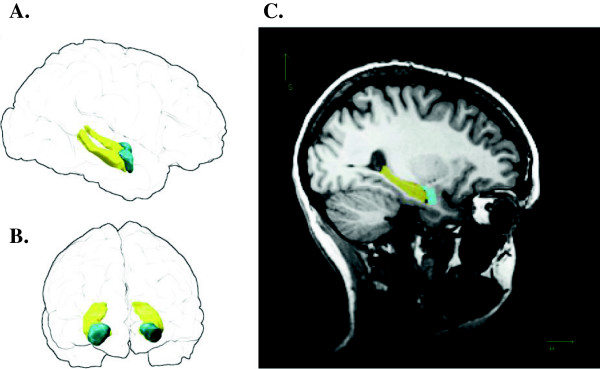
**Parcellation of hippocampus and amygdala in a representative participant (female, right hippocampal volume ranked 15/30).** Left: “glass brain” renderings showing three-dimensional volumes of right and left hippocampi (yellow) and right and left amygdalae (cyan) viewed from participant’s right **(A)** and front **(B)**; the outline of the pial surface is shown in black. **C**. Right labelled voxels overlaid on a T1 image, sagittal section passing through right hippocampus (yellow) and amygdala (cyan).

T1-weighted structural MRI images were obtained from our participants at the York Neuroimaging Centre on a GE 3 T HD Excite MRI Scanner (General Electric Medical Systems, Milwaukee, WI). Whole-brain T1-weighted data sets were acquired in the sagittal plane using fast spoiled gradient reaction echo (3DFSPGR) sequence to collect data from 176 continuous slices (repetition time = 7.8 ms, echo time = 3 ms, inversion time = 450 ms, field of view = 290 × 290 × 176, matrix size = 256 × 256 × 176, slice thickness = 1.0 mm, resolution = 1.13 × 1.1.3 × 1.0 mm, flip angle = 20°)^a^. Automated subcortical and cortical segmentation was performed using Freesurfer version 5.1 [44]. Parcellation of the subcortical and cortical anatomy, and calculations of the total subcortical gray matter volume, total gray matter volume and intracranial volume were performed by delineating anatomical divisions via FreeSurfer’s automatic parcellation methods, in which the statistical knowledge base derives from a training set incorporating the anatomical landmarks and conventions described by Duvernoy [45]. This procedure assigns a neuroanatomical label to each voxel in an MRI volume based on probabilistic information estimated from a manually labelled training set. This classification technique employs a non-linear registration procedure that is robust to anatomical variability [46]. The segmentation uses three pieces of information to disambiguate labels: (1) the prior probability of a given tissue class occurring at a specific atlas location; (2) the likelihood of the image given what tissue class; and (3) the probability of the local spatial configuration of labels given the tissue class. The technique has shown comparable accuracy to manual labelling [46]. The hippocampus and amygdala were identified as regions of interest based on previous literature on the neural bases of anxiety [5]. This, as well as volumes (mm^3^) for total subcortical gray matter volume, total gray matter volume and intracranial volume, were obtained from the statistics output file (aseg.stats). An example of the parcellation results is shown for a representative participant in Figure 1.

### Data analysis

On initial analysis of the data we found two major consistent predictors of hippocampal volume (age and sex), which can potentially confound estimation of the relationship of hippocampal volume with anxiety traits. Previous developmental studies of hippocampal volume show that hippocampal volume peaks in middle age (approximately 45 years, [47]). Consistent with these findings, age had a positive significant correlation with hippocampal volume in our young sample (Pearson’s r, total hippocampal volume versus age, r(30) = 0.41, *P* = 0.025) and men were found to have larger hippocampi than women (bilateral raw hippocampal volume, independent t-test, two-tailed, t(28) = -2.72, *P* = 0.011). This was also the case for the amygdala; age had a positive correlation with amygdala volume (Pearson’s r = 0.36 *P* = 0.051), and men were found to have larger amygdala volume than women (bilateral raw amygdala volume, independent t-test, two-tailed, t(28) = -4.02, *P* = 0.001). In addition, we found a general effect of sex on brain volume such that, in comparison to females, males had greater total gray matter volume (t(28) = 4.374, *P* = 0.001), total subcortical gray matter volume (t(28) = 4.315, *P* = 0.001), and intracranial volume (t(28) = 3.394, *P* = 0.002).

In order to establish the extent to which StP predicted brain volumes, and to control for sex- and age-related potential confounds mentioned above, we incorporated intracranial volume, age, and sex as co-regressors alongside StP into multiple regression models. All the beta values that we report are standardised beta values. A recent methodological study [48] addressing volume correction in structural MRI studies has specifically recommended the use of intracranial volume, age, and sex as covariates in multiple regression models relating variables of interest to specific brain region volumes. To investigate effects of hippocampal lateralisation, we calculated a laterality index specific to that structure using the formula: Right - Left hippocampal volume)/total hippocampal volume; that is, a unit-less measure. One advantage of this measure is that it obviates the need for co-regressors controlling for whole volume. Essentially, this measure sacrifices information about the absolute volume of each hippocampus in order to obtain a well-controlled measure of laterality. Analysis using this measure makes fewer assumptions about linearity and stability of association between variables. For instance, the average relationship between hippocampal volume and intracranial volume may not be expected to be constant across a sample of different ages. Each analytic approach yielded convergent results regarding the association between right hippocampal volume and Gray’s BIS as indexed by the StP scale. All statistical analyses were conducted using SPSS version 20.0 (SPSS Inc., Chicago, IL, USA).

## Results

### Participant characteristics

Participant demographics and self-report measure scores are summarized in Table 1. StP scores did not differ between males and females in the sample (independent t-test, two-tailed t(28) = 1.12, *P* = 0.27). There were also no gender differences in STAI-T scores in this sample (independent t-test, two-tailed, t(28) = 1.36, *P* = 0.18). Since the Shapiro-Wilks test indicated that BAI, BDI and negative LES scores were not normally distributed, they were analysed using the Mann–Whitney test. This analysis revealed a gender differences in BAI and negative LES scores, where females had higher BAI scores, and reported a greater number of negative life events (BAI, U = 47.50, z = -2.70, *P* = 0.007; negative life events, U = 43.50, z = -2.86, *P* = 0.004). There were no gender differences in scores for depression as measured by BDI (U = 84.00, z = -1.17, *P* = 0.24).

**Table 1:** Participants demographics and self-report measure scores

	**Female**		**Male**		**Total**	
	**Range**	**Mean (SD)**	**Range**	**Mean (SD)**	**Range**	**Mean (SD)**
Age	20-27	23.29 (1.73)	22-32	24.81 (3.15)	20-32	24.10 (2.66)
Intelligence quotient	100-129	117.86 (9.04)	90-131	118.5 (10.15)	90-131	118.20 (9.48)
Life experiences survey positive	3-29	12.93 (7.91)	3-24	16.19 (6.71)	3-29	14.67 (7.35)
Life experiences survey negative	2-27	9.86 (6.87)	1-11	4.38 (2.73)	1-27	6.93 (5.72)
Beck depression inventory	0-18	4.86 (4.91)	0-8	2.69 (2.18)	0-18	3.70 (3.81)
Beck anxiety inventory	5-20	9.43 (4.72)	0-17	5.00 (4.63)	0-20	7.07 (5.11)
Trait anxiety	44-79	54.64 (9.95)	37-65	50.13 (8.22)	37-79	52.23 (9.20)
Sensitivity to Punishment	1-16	9.29 (4.97)	2-19	7.38 (4.41)	1-19	8.27 (4.70)

### Volumetric brain measures

Bilateral, right and left hippocampal and amygdala volumes (Figure 1), and intracranial volume, total gray matter volume, and subcortical gray matter volumes are shown in Table 2. In this study our specific aim was to investigate the relationship between hippocampal volume and behavioural inhibition. One potentially important predictor of hippocampal volume is depression; however, in our healthy sample we found no evidence that depression scores co-varied with hippocampal volume (Spearman’s rho = 0.034, *P* = 0.858).

**Table 2:** Volumetric brain measures

	**Female**		**Male**		**Total**	
**Volume (mm**^**3**^**)**	**Range**	**Mean (SD)**	**Range**	**Mean (SD)**	**Range**	**Mean (SD)**
Intracranial volume	1,066,529.5-1,581,389.1	1,365,722.9 (187,852.801)	1,079,697.4-1,945,463.8	1,628,006.319 (229,462.0061)	1,066,529.5-1,945,463.8	1,505,607.39 (246,506.369)
Total gray matter	576,015-735,158	652850 (49,290)	657,592.1-875,048	739,810 (59,634)	576,015-875,048	699,230 (69,827)
Subcortical gray matter	156,112-210,381	182988 (14,859)	185,983-252,345	209,839 (18,269)	156,112-252,345	197,309 (21,383)
Bilateral hippocampus	8,083-10,763	8,860 (775)	7,786-11,605	9,826 (1,114)	7,786-11,605	9,375 (1,073)
Right hippocampus	3,568-5,345	4,395 (430)	3,814-5,688	4,760 (551)	3,568-5,688	4,590 (524)
Left hippocampus	3,993-5,418	4,464 (417)	3,972-6,150	5,065 (613)	3,972-6,150	4,785 (604)
Bilateral amygdala	2,443-3,282	2,777 (236)	2,427-4,240	3,266 (397)	2,427-4,240	3,038 (410)
Right amygdala	1,294-1,660	1,442 (105)	1,404-2,248	1,663 (194)	1,294-2,248	1,560 (192)
Left amygdala	1,065-1,622	1,334 (152)	1,023-2,113	1,603 (236)	1,023-2,113	1,478 (240)

### Right hippocampal volume is correlated with Sensitivity to Punishment

Using multiple regression to examine the relationship between right hippocampal volume and StP, we controlled for age, sex, and intracranial volume (ICV) by including these variables as co-regressors alongside StP scores. A significant model emerged (F(4,29) = 4.789, *P* = 0.005, adjusted R^2^ = 0.343), whereby StP (β = 0.334, *P* = 0.040) and age (β = 0.379, *P* = 0.024) but not the other variables (sex, β = -0.148, *P* = 0.440; ICV, β = 0.305, *P* = 0.10) predicted right hippocampal volume.

Furthermore, we ran an additional analysis using a two-step approach in a hierarchical regression model, where step 1 included sex, age and ICV, and step 2 added StP. This analysis showed that StP explains a further 11% of the variance in right hippocampal volume over and above the initial model including sex, age and ICV, and the significance of the change in F from the first to second model was *P* = 0.04 (step 1, R^2^ change = 0.327; step 2, R^2^ change = 0.107).

### Right/left hippocampal laterality is correlated with Sensitivity to Punishment

**Figure 2:**
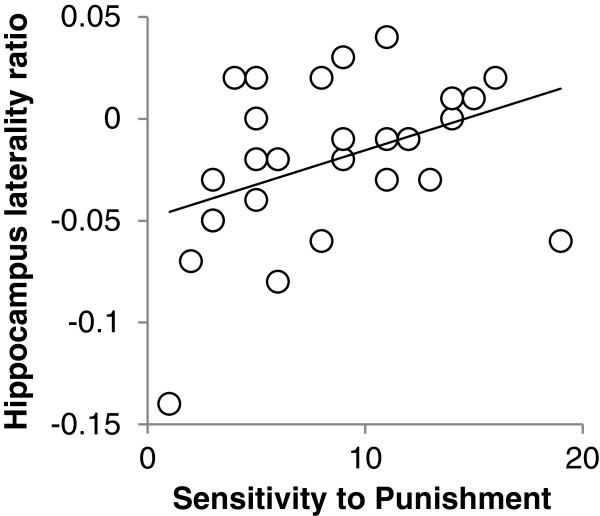
**Relationship between right/left hippocampal laterality and StP scores.** Individuals where the right hemisphere may be approaching symmetry with the left or overtaking it in terms of size have higher scores on the Sensitivity to Punishment scale suggestive of a more hyperactive Behavioural Inhibition System. Hippocampus laterality ratio = (right - left hippocampal volume)/total hippocampal volume; a zero score would reflect a perfectly symmetric hippocampus.

To investigate the right hippocampus laterality effect further, we next calculated a ratio measure of hippocampal laterality, by dividing the right minus the left hippocampal volume by the total hippocampal volume, where a zero score would reflect a perfectly symmetric hippocampus (laterality ratio scores: range = -0.14 to 0.04; mean = -0.02 SD = 0.038). One advantage of this measure is that it obviates the need for co-regressors controlling for whole volume (see Methods). We performed a multiple regression analysis controlling for age and sex by including these variables as co-regressors alongside hippocampal laterality scores. Using this approach, a significant model emerged (F(3,29) = 3.238, *P* = 0.038, adjusted R^2^ = 0.188), where only StP significantly predicted right/left hippocampal laterality (StP, β = 0.383, *P* = 0.034; age, β = 0.211, *P* = 0.239; sex, β = 0.285, *P* = 0.122). Furthermore, we ran an additional analysis using a two-step approach in a hierarchical regression model, where step 1 included sex, age and ICV, and step 2 added StP. This analysis showed that StP explains a further 15% of the variance in right/left hippocampal laterality, over and above the initial model (step 1), with the significance of the change in F from the first to second model being *P* = 0.019 (step 1, R^2^ change = 0.248; step 2, R^2^ change = 0.151, StP β = 0.397). For illustrative purposes, Figure 2 shows the relationship between right/left hippocampal laterality and StP scores.

### Sensitivity to Punishment was not significantly correlated with either left hippocampal or amygdala volumes

When we replaced the volume of the right hippocampus in the multiple regression analysis with the volume of the left hippocampus or either the left or right amygdala, again controlling for known associations of age, sex, and ICV, we found no relationship between these regions and StP (Additional file 2: Table S2).

### No significant relationship between hippocampal volume and other anxiety constructs

Although our focus was on the animal literature-based behavioural inhibition approach to anxiety conceptualised by Gray, and implemented through the StP instrument of Torrubia and colleagues [16], we also examined whether the relationship observed between right hippocampal volume and StP was specific to the BIS construct of anxiety, or whether a similar relationship existed for other constructs of anxiety. Using the same regressions performed for the StP scores, we asked to what extent hippocampal volume could be predicted by two additional and well-established anxiety constructs, STAI-T and BAI. One multiple regression analysis was run with STAI-T and a second multiple regression analysis with BAI (both controlling for age, sex, and ICV). These revealed that neither STAI-T nor BAI predicted either right or left hippocampal volumes (right hippocampus: STAI-T, β = 0.215, *P* = 0.205; BAI, β = 0.114, *P* = 0.580; left hippocampus: STAI-T, β = 0.199, *P* = 0.172; BAI, β = -0.0110 *P* = 0.953; for the other relationships see Additional file 3: Table S3). To gain some idea of the overlap and specificity of these measures, we tested for correlations among the three different anxiety measures (StP, STAI-T, BAI) and the BDI (Table 3). We note that, firstly, STAI-T scores were, but StP and BAI scores were not, significantly correlated with depression scores and, secondly, that StP scores were significantly correlated with STAI-T scores, but not with BAI scores.

**Table 3:** Correlations between self-report inventories of depression and anxiety

**Measure**	**BDI**	**BAI**	**STAI-T**
**Beck Depression Inventory (BDI)**	-		
**Beck Anxiety Inventory (BAI)**	0.266	-	
**Trait anxiety of the State and Trait Anxiety Inventory (STAI-T)**	0.490^**^	0.446^*^	-
**Sensitivity to Punishment**	0.306	0.341	0.536^**^

Spearman’s rho (n = 30) two-tailed; **P < 0.01, *P < 0.05.

## Discussion

In this study we examined whether a relationship exists between hippocampal volume and behavioural inhibition, as measured by the StP scale. We found that high scores on the StP scale were positively associated with hippocampal volume, when controlling for both sex, age and ICV, and that this phenomenon was lateralized to the right side.

### The Behavioural Inhibition System, anxiety, and the hippocampus

Our findings contribute to a growing body of work showing that the hippocampus plays a critical role in anxiety-related behaviour as part of the BIS [6]. Including our own, there are now three studies that show a positive relationship between hippocampal volume and BIS activity in non-clinical populations [21,22]. These results lend support to Gray’s theory of the neurobiological basis of anxiety [4]. However, although motivated by pre-existing theory, such correlational findings cannot directly suggest whether such anatomical variations precede or follow from the behavioural, cognitive and affective effects of BIS related activity. Indeed, it seems possible that both genetic and experiential factors as well as their interactions may contribute to the observed association. Genetic factors are very likely to be important. For instance, while both hippocampal and amygdalar activity (as measured by positron emission tomography imaging) predicted behavioural inhibition in a study on monkeys, only hippocampal activity was found to be heritable [20]. Although gray matter volume in the hippocampus is not as strongly genetically determined as it is in regions such as the lateral prefrontal cortex, its heritability still appears to be moderate to high, at 40 to 69% [49].

That experiential factors are important is suggested by human longitudinal structural neuroimaging studies, which show that repeated activation of a brain region, either whilst learning new skills [50-52] or through transcranial magnetic stimulation [53], can lead to an increase in the corresponding region’s gray matter volume. Thus, it is plausible that the increased gray matter volume we observed in the right hippocampus may reflect an increase in activity of this region, associated with higher levels of BIS-based anxiety. Further, the positive relationship between BIS activity and hippocampal volume observed in this study and by others complements neuroimaging studies that have found that BIS-related measures are associated with greater activation of the hippocampus to aversive stimuli [54,55]. Moreover, consistent with the right-side relationship between BIS and hippocampal volume we found in this study, Mathews and colleagues [54] found that enhanced activation by fear-related versus neutral pictures was more pronounced in those individuals with high BIS scores specifically in the right hippocampus.

Interestingly, the correlation we and others report regarding BIS and hippocampal volume is a positive correlation. A classic problem of investigating anxiety in clinical populations is that it is often associated with depression. Estimates reported in Van Tol and colleagues [56] indicate that the comorbidity of anxiety disorders and depression ranges from 10% to over 50%, and have shown that major depressive episodes are associated with a significantly smaller gray matter volume of both the hippocampus and amygdala. Notably, other pathologies, such as seen in psychopathy [57] and schizophrenia [58], are often associated with smaller hippocampi. Since anxiety may often precede depression [59], it remains possible that smaller hippocampal and amygdalar volumes primarily occur after depression sets in. Some studies have shown reduced hippocampal volume in post-traumatic stress disorder (PTSD) [60,61]. PTSD is associated with high levels of trauma and stress, both of which are known to increase levels of corticosteroids [62,63] which in turn reduce both amygdalar [64] and hippocampal volumes [65,66]. Some researchers [67] have argued that trauma, rather than anxiety or PTSD *per se*, is associated with smaller gray matter volume, supported by their study of severe burn victims without PTSD who had significantly smaller hippocampal volumes than patients with no experience of trauma [67]. Notably, StP, unlike STAI-T, was not significantly correlated with depression scores on the BDI in our sample.

In all, this suggests that different aspects of anxiety may have dissociable and potentially opposing relationships with hippocampal volume. Our observation of increased hippocampal volume in BIS anxiety may have been facilitated by our relatively restricted sample - young, well-educated people who had not experienced many negative and stressful life events. Although it was not the main focus of our study, we note that STAI-T measure and anxiety measured by BAI were not significantly positively correlated with hippocampal volume while the BIS anxiety measure was. We caution against prematurely interpreting this as a dissociation, but this would be consistent with the view that different anxiety scales measure somewhat different forms of anxiety or negative emotionality, with potentially distinct neurobiological bases, and that a multidimensional rather than unitary approach to anxiety is appropriate. For instance, it has been suggested that the STAI-T measure may predict depression and negative affect as much as, or even more than, anxiety *per se*[36,37]. Consistent with this we also found that the STAI-T measure was highly positively correlated with BDI.

### Brain laterality effects and the Behavioural Inhibition System

Our findings suggest that aspects of anxiety associated with the BIS may be lateralized to the right hemisphere, and/or depend on the relative asymmetry of the left and right hippocampus. Interestingly, a number of studies of individuals with severe psychiatric disorders have found that asymmetry of the hippocampus is normative, whilst symmetry is not [68-70]. In our sample, we found overall that the left hippocampus was larger than the right; hence, our hippocampal laterality ratio indicates that participants where the right hemisphere may be approaching symmetry with the left or overtaking it in terms of size may have a more hyperactive BIS. Consistent with this, some researchers have suggested that some aspects of anxiety may be lateralized to the right hemisphere [71-73], and heightened right hemisphere activity [74], and structural changes [75] in general has been reported for clinical anxiety populations.

### The Behavioural Inhibition System and the amygdala

Three studies, including ours, that could have observed a relationship between amygdala volume and BIS activity did not find any such relationship [21,23]. To our knowledge, one study to date, Barros-Loscertales and colleagues [22], has found a positive relationship between amygdala volume and StP scores (using VBM analysis). We have no obvious explanation for these differences, but note that we, like Barros-Loscertales and colleagues [22], did observe a positive correlation between StP scores and hippocampal volume. Whether this implies that the association between the BIS and hippocampal volume is more reliable (and perhaps more heritable) than that between the BIS and amygdala remains speculative at this point, and deserving of study. Further studies would be required to investigate if there is a difference between hippocampal and amygdalar volume relationships to StP, as would be consistent with the view of Gray and McNaughton [5] that they contribute differently to anxiety. Briefly, for instance, these authors posit that the hippocampus plays a greater role in behavioural inhibition and risk assessment aspects of anxiety, while the amygdala plays a greater role in increased arousal and active avoidance. It must be noted that our sample was relatively small (n = 30). Importantly, then, we cannot rule out the possibility that a larger number of participants might have revealed an association between StP scores and amygdala volumes. Because of this, we would caution against interpreting our findings as positive evidence of the lack of an association between amygdala volume and StP.

### Limitations of study

It is worth noting that a limitation of our study was our relatively small sample size, which was also restricted to young, well-educated people, which might limit the generalisability of the results. Therefore, replication of our results using larger samples is necessary. Our focus was on Gray’s conception of anxiety, based originally on the role of the hippocampus in behavioural inhibition as seen in the animal literature, including the highly replicable observation that anxiolytic drugs reliably disrupt not only behavioural inhibition but also hippocampal theta. Notably, we have recently extended this observation to freely-moving animals [14]. Thus, our focus was on StP, an instrument designed specifically to assess Gray’s construct of behavioural inhibition. We compared results using StP to two other standard measures of anxiety (STAI-T and BAI) for illustrative purposes, but did not perform a correction for multiple comparisons. In our sample, StP scores were significantly correlated with STAI-T scores, but not BAI scores or BDI scores. We caution that these are only suggestive hints of the potential selectivity of the StP measure and its positive correlation with hippocampal volume. Larger studies and meta-analyses will be required to definitively disentangle shared and separate contributions to anxiety, and to incorporate any direct and secondary effects relating to stress and depression.

Larger studies should also examine the possibility that there may be interactions between sex and other variables, which our study is underpowered to detect. For instance, it remains a possibility that associations between hippocampal volume measures and StP vary between sexes, and/or that these associations are related to age. Our results clearly point to an association between right hippocampal volume and StP in our sample. However, our limited methodology did not allow us to test whether a larger absolute volume of the right hippocampus is most predictive of StP scores, or rather, a relatively large right hippocampus with respect to the left hippocampus, conceivably reflecting a tendency away from left hemispheric dominance towards right hemispheric dominance which has previously been associated with measures of Gray’s BIS [24,25,30].

## Conclusions

We investigated the relationship between participants’ self-report of behavioural inhibition and the volume of two brain regions, the hippocampus and amygdala, previously linked to anxiety in research in rodents, non-human primates and humans. Behavioural inhibition was measured by the StP scale designed to implement Gray’s construct of anxiety. Brain volume was measured by structural MRI using FreeSurfer’s automatic segmentation method to obtain gray matter volume estimates of the hippocampus and amygdala. Results revealed a positive association between behavioural inhibition and right hippocampal volume. These findings suggest that structural variation or change affecting hippocampal volume, and the relative size of left and right hippocampi in particular, may reflect a predisposition to, or play a part in mediating BIS-related anxiety, and support the idea that morphological differences in the hippocampal formation may reflect a risk factor for developing anxiety.

## Endnote

^a^One participant was scanned using a different protocol with 1.0 × 1.0 mm in-plane resolution, repetition time = 8.06 ms; echo time = 3 ms; inversion time = 600 ms; flip angle = 12°.

## Abbreviations

BAI: Beck Anxiety Inventory; BDI: Beck Depression Inventory; BIS: Behavioural Inhibition System; EEG: electroencephalography; ICV: intracranial volume; IQ: intelligence quotient; LES: Life Experiences Survey; MRI: magnetic resonance imaging; PTSD: post-traumatic stress disorder; STAI-T: Trait anxiety of the State and Trait Anxiety Inventory; StP: Sensitivity to Punishment; VBM: voxel-based morphometry.

## Competing interests

The authors declare that they have no competing interests.

## Authors’ contributions

LL, TH and CL designed the study and performed the statistical analysis, and were involved in the writing of the manuscript. CB made a significant contribution to the laterality analysis and first draft. CB, AH, ES and EP collected the data, contributed to the initial data analysis and to the first draft of the paper. LL wrote the first draft of the manuscript. All authors read and approved the final manuscript.

## Supplementary Material

Additional file 1: Table S1 Correlations of psychometric measures with age. Click here for file

Additional file 2: Table S2 Multiple regression to examine the relationship between left hippocampal volume and StP, and the left and right amygdala volumes and StP. Click here for file

Additional file 3: Table S3 Multiple regression to examine the relationship between right and left hippocampal volumes and anxiety as measured by STAI-T and BAI. Click here for file
