# Supplementary material for: The Behavioural Inhibition System, anxiety and hippocampal volume in a non-clinical population
Source: Biol Mood Anxiety Disord. 2014 Mar 7;4:4. doi: 10.1186/2045-5380-4-4 (PMC4007806; doi:10.1186/2045-5380-4-4)
Supplement: Additional file 1: Table S1 — Correlations of psychometric measures with age. [file 2045-5380-4-4-S1.docx]

**Table S1**

|  |  | Age |
| --- | --- | --- |
| I.Q | Spearman's rho | 0.068 |
|  | Sig. (2-tailed) | 0.719 |
|  | N | 30 |
| Sensitivity to Punishment | Spearman's rho | 0.035 |
|  | Sig. (2-tailed) | 0.855 |
|  | N | 30 |
| Trait Anxiety | Spearman's rho | -0.049 |
|  | Sig. (2-tailed) | 0.798 |
|  | N | 30 |
| Beck Anxiety Inventory | Spearman's rho | 0.415* |
|  | Sig. (2-tailed) | 0.022 |
|  | N | 30 |
| Beck Depression Inventory | Spearman's rho | -0.122 |
|  | Sig. (2-tailed) | 0.516 |
|  | N | 30 |
| LES positive | Spearman's rho | 0.270 |
|  | Sig. (2-tailed) | 0.149 |
|  | N | 30 |
| LES negative | Spearman's rho | -0.165 |
|  | Sig. (2-tailed) | 0.384 |
|  | N | 30 |

***p < 0.05**
