# Supplementary material for: The Behavioural Inhibition System, anxiety and hippocampal volume in a non-clinical population
Source: Biol Mood Anxiety Disord. 2014 Mar 7;4:4. doi: 10.1186/2045-5380-4-4 (PMC4007806; doi:10.1186/2045-5380-4-4)
Supplement: Additional file 2: Table S2 — Multiple regression to examine the relationship between left hippocampal volume and StP, and the left and right amygdala volumes and StP. [file 2045-5380-4-4-S2.docx]

**Table S2**

| **Dependent Variable: Left hippocampus volume** | | | | | | | | | | | |
| --- | --- | --- | --- | --- | --- | --- | --- | --- | --- | --- | --- |
|  | | |  | |  | |  | |  | | |
| **Predictors** | | **β** | | **p** | | **Model** | |  | | |  |
|  | |  | |  | | **Adjusted R2** | | 0.426 | | |  |
| **StP** | | 0.085 | | 0.560 | | **F(4, 29)** | | 6.389 | | |  |
| **ICV** | | 0.533 | | 0.004 | | **p** | | 0.001 | | |  |
| **Age** | | 0.213 | | 0.160 | |  | |  | | |  |
| **Sex** | | -0.173 | | 0.336 | |  | |  | | |  |
|  |  | |  | |  | |  | | |  |  |

| **Dependent Variable: Right amygdala volume** | | | | | | | | | |  |
| --- | --- | --- | --- | --- | --- | --- | --- | --- | --- | --- |
|  | | |  | |  | |  | |  |  |
| **Predictors** | | **β** | | **p** | | **Model** | |  |  |  |
|  | |  | |  | | **Adjusted R2** | | 0.511 |  |  |
| **StP** | | 0.006 | | 0.964 | | **F(4, 29)** | | 8.570 |  |  |
| **ICV** | | 0.578 | | 0.001 | | **p** | | 0.001 |  |  |
| **Age** | | 0.445 | | 0.660 | |  | |  |  |  |
| **Sex** | | -0.253 | | 0.132 | |  | |  |  |  |
|  |  | |  | |  | |  | |  | |
| **Dependent Variable: Left amygdala volume** | | | | | | | | | |  |
|  | | |  | |  | |  | |  |  |
| **Predictors** | | **β** | | **p** | | **Model** | |  |  |  |
|  | |  | |  | | **Adjusted R2** | | 0.637 |  |  |
| **StP** | | -0.60 | | 0.606 | | **F(4, 29)** | | 13.697 |  |  |
| **ICV** | | 0.635 | | 0.001 | | **p** | | 0.001 |  |  |
| **Age** | | 2.555 | | 0.017 | |  | |  |  |  |
| **Sex** | | -0.882 | | 0.386 | |  | |  |  |  |
|  |  | |  | |  | |  | |  | |
