# Supplementary material for: The Behavioural Inhibition System, anxiety and hippocampal volume in a non-clinical population
Source: Biol Mood Anxiety Disord. 2014 Mar 7;4:4. doi: 10.1186/2045-5380-4-4 (PMC4007806; doi:10.1186/2045-5380-4-4)
Supplement: Additional file 3: Table S3 — Multiple regression to examine the relationship between right and left hippocampal volumes and anxiety as measured by STAI-T and BAI. [file 2045-5380-4-4-S3.docx]

**Table S3**

| **Dependent Variable: Right hippocampus volume** | | | | | | | | | |  |
| --- | --- | --- | --- | --- | --- | --- | --- | --- | --- | --- |
|  | | |  | |  | |  | |  |  |
| **Predictors** | | **β** | | **p** | | **Model** | |  |  |  |
|  | |  | |  | | **Adjusted R2** | | 0.269 |  |  |
| **Trait anxiety** | | 0.215 | | 0.205 | | **F(4, 29)** | | 3.666 |  |  |
| **ICV** | | 0.302 | | 0.123 | | **p** | | 0.018 |  |  |
| **Age** | | 0.362 | | 0.039 | |  | |  |  |  |
| **Sex** | -0.138 | | 0.499 | |  | |  | |  | |
|  | |  | |  | |  | |  |  |  |
| **Dependent Variable: Left hippocampus volume** | | | | | | | | | |  |
|  | | |  | |  | |  | |  |  |
| **Predictors** | | **β** | | **p** | | **Model** | |  |  |  |
|  | |  | |  | | **Adjusted R2** | | 0.461 |  |  |
| **Trait anxiety** | | 0.199 | | 0.172 | | **F(4, 29)** | | 7.201 |  |  |
| **ICV** | | 0.520 | | 0.004 | | **p** | | 0.001 |  |  |
| **Age** | 0.198 | | 0.178 | |  | |  | |  | |
| **Sex** | | -0.216 | | 0.224 | |  | |  |  |  |
|  | |  | |  | |  | |  |  |  |
|  | |  | |  | |  | |  |  |  |
|  |  | |  | |  | |  | |  | |
| **Dependent Variable: Right hippocampus volume** | | | | | | | | | |  |
|  | | |  | |  | |  | |  |  |
| **Predictors** | | **β** | | **p** | | **Model** | |  |  |  |
|  | |  | |  | | **Adjusted R2** | | 0.229 |  |  |
| **BAI** | | 0.114 | | 0.580 | | **F(4, 29)** | | 3.153 |  |  |
| **ICV** | | 0.336 | | 0.098 | | **p** | | 0.031 |  |  |
| **Age** | | 0.331 | | 0.092 | |  | |  |  |  |
| **Sex** | -0.126 | | 0.578 | |  | |  | |  | |
|  | |  | |  | |  | |  |  |  |
| **Dependent Variable: Left hippocampus volume** | | | | | | | | | |  |
|  | | |  | |  | |  | |  |  |
| **Predictors** | | **β** | | **p** | | **Model** | |  |  |  |
|  | |  | |  | | **Adjusted R2** | | 0.418 |  |  |
| **BAI** | | -0.11 | | 0.953 | | **F(4, 29)** | | 6.126 |  |  |
| **ICV** | | 0.535 | | 0.004 | | **p** | | 0.001 |  |  |
| **Age** | 0.217 | | 0.199 | |  | |  | |  | |
| **Sex** | | -0.148 | | 0.453 | |  | |  |  |  |
|  | |  | |  | |  | |  |  |  |
|  | |  | |  | |  | |  |  |  |
|  | |  | |  | |  | |  |  |  |
|  | |  | |  | |  | |  |  |  |
|  |  | |  | |  | |  | |  | |
